# Vitamin P Significantly Inhibits the Expression of Ki67 and VEGF and Promotes the Apoptosis of Colorectal Cancer Cells

**DOI:** 10.5812/ijpr-163955

**Published:** 2026-02-21

**Authors:** Xin Zhao, Shang Guo, Rongwei Shen, Wei Zhang, Yamin Zhang

**Affiliations:** 1 First Central Hospital of Tianjin Medical University, Tianjin, China; 2 Tianjin First Central Hospital, Tianjin, China; 3 Sun Yat-sen University First Affiliated Hospital, Guangzhou, China

**Keywords:** Ki67, Vascular Endothelial Growth Factor (VEGF), Vitamin P, Colorectal Cancer, Apoptosis

## Abstract

**Background:**

Colorectal cancer is a common malignant tumor of the digestive tract, with a high incidence and mortality rate. Ki67 and vascular endothelial growth factor (VEGF) play important roles in tumor cell proliferation and angiogenesis. Vitamin P is a natural flavonoid compound with various biological activities.

**Objectives:**

To explore the effects of vitamin P on the proliferation, angiogenesis, and apoptosis of colorectal cancer cells and its underlying molecular mechanisms.

**Methods:**

In vitro cultivation of human colorectal cancer cell lines (HCT116 and SW480) was performed. Vitamin P was added to the cells in varying quantities. Cell proliferation was identified using the CCK-8 technique. The flow cytometry method was used to determine the proportion of apoptotic cells. Real-time fluorescence quantitative PCR and Western blot methods were used to measure the expression levels of the VEGF and Ki67 genes and proteins. Secretion of VEGF was observed via immunofluorescence staining.

**Results:**

Vitamin P treatment significantly inhibited the proliferation of colorectal cancer cells in a dose-dependent manner. The half-maximal inhibitory concentrations (ICs_50_) at 48 hours were 72.3 μM (HCT116) and 85.6 μM (SW480). Moreover, in the vitamin P treatment group (42.7 - 68.9% and 35.4 - 61.2%), the secretion of VEGF decreased by 52.3 - 79.8%. The percentage of apoptotic cells induced by 20 - 80 μM vitamin P increased from 5.3% in the control group to 18.6 - 37.9%, with increased caspase-3 activity. In vivo experiments showed that vitamin P significantly inhibited the growth of colorectal cancer transplanted tumors and reduced the expression of Ki67 and VEGF in the tumors. Meanwhile, vitamin P treatment induced apoptosis of tumor cells, and this effect was closely related to the regulation of the Bax/Bcl-2 ratio and the promotion of caspase-3 activation. Vitamin P may play a regulatory role by inhibiting the phosphorylation of the STAT3 signaling pathway and downregulating the expression of proteins related to the PI3K/AKT pathway.

**Conclusions:**

Vitamin P can inhibit the malignant biological behavior of colorectal cancer cells by suppressing Ki67-mediated cell proliferation, blocking VEGF-related angiogenesis pathways, and activating mitochondrial apoptotic pathways.

## 1. Background

Colorectal cancer occurrence and development are closely related to abnormal cell proliferation, an imbalance in angiogenesis, and the inhibition of apoptosis (1). Although significant progress has been made in clinical regimens such as surgery combined with chemotherapy and targeted therapy, tumor drug resistance and treatment side effects remain urgent problems to be solved (2-4). In recent years, natural products have gradually become a hot topic in the research of antitumor drugs because of their multitarget action characteristics and the advantage of low toxicity (5). Vitamin P, a flavonoid compound widely present in plants, has been proven to have anti-inflammatory, antioxidant, and antitumor activities, but its specific mechanism of action in colorectal cancer has not been fully clarified (6). However, systematic studies on the expression of the proliferation markers Ki67 and vascular endothelial growth factor (VEGF) and apoptotic pathways in colorectal cancer cells are still insufficient. Notably, the abnormal expression of Ki67, whereas VEGF-mediated angiogenesis is an important link in the formation of the tumor microenvironment (7-9). At present, although drugs targeting VEGF have been applied in clinical practice, their drug resistance and specificity still need to be optimized (10-12). 

## 2. Objectives

This study focused on exploring the molecular mechanisms by which vitamin P regulates proliferation and angiogenesis. By integrating the latest progress in domestic and international research on the anticolorectal cancer effects of natural products and combining experiments to verify the effects of vitamin P on the expression of Ki67 and VEGF proteins and apoptosis-related proteins in the SW480/HCT116 cell model. This study not only helps reveal the multidimensional antitumor effects of vitamin P but also provides a theoretical foundation for creating adjuvant therapy plans for colorectal cancer using natural products.

## 3. Methods

### 3.1. Main Reagents and Instruments

Vitamin P (purity 98.6%) (Selleck, USA), DMEM, fetal bovine serum (FBS) (Invitrogen, USA), a streptomycin mixture (100×), a CCK-8 kit, a glyceraldehyde 3-phosphate dehydrogenase goat anti-rabbit IgG labeled with a GAPDH antibody, and a horseradish peroxidase (HRP) (Abcam, UK) were used. An MQX-200 microplate reader (Bio-Tek, USA), a Calibur flow cytometer (BD, USA), and an IX71 inverted fluorescence microscope (Olympus, Japan) were used. This study has been approved by the Medical Research Animal Ethics Committee [NO. HKYS-2025-A0261].

### 3.2. Experimental Methods

#### 3.2.1. CCK-8 Experiment

A density of 5 × 10^3^ cells/mL was used to seed HCT116 and SW480 cells in 96-well plates. Vitamin P (dissolved in DMSO) at concentrations of 0, 5, 10, 20, 40, 80, and 160 μmol/L was used to cultivate the cells. The samples were incubated for two hours at 37°C after ten microliters of CCK-8 solution were added to each well. A microplate reader was used to read the absorbance values of each well.

#### 3.2.2. Cloning Formation Experiments

In 6-well plates, 800 HCT116 and SW480 cells were seeded per well, and the plates were then grown in an incubator. Then, they were cultured with 0, 10, 20, or 40 μmol/L vitamin P for 14 days. Cell clones (containing 50 or more cells as one clone) were observed and counted under an inverted fluorescence microscope, and the colony formation rate was calculated.

#### 3.2.3. Western Blotting

After being exposed to 0, 10, 20, or 40 μmol/L of vitamin P for a whole day, anti-Ki67, anti-Bcl-2, and anti-Bax antibodies were added, and the samples were cultured overnight at 4°C. After adding goat anti-rabbit IgG tagged with HRP, the samples were incubated for an hour at 37°C. The ChemiDoc MP imaging device was used to take pictures of the protein bands, and ImageJ software was used to assess the protein bands' gray values.

#### 3.2.4. Flow Cytometry

After being exposed to vitamin P at concentrations of 0, 10, 20, and 40 μmol/L for a full day, the cells were rinsed twice with cooled PBS, suspended once more, and centrifuged for five minutes at 12,000 ×g. The cells were allowed to sit at room temperature for 10 minutes in the dark. The degree of cell apoptosis was analyzed via flow cytometry.

#### 3.2.5. Scratch Closure Test

HCT116 and SW480 cells were inoculated into 6-well plates (3×10^5^ cells per well) and cultured for 24 hours. After the cells were adherent, a 200 μL pipette was used to create a vertical scratch in the cell culture plate along the diameter of the 6-well plate. The culture medium was discarded, and fresh culture medium containing 0, 10, 20, or 40 μmol/L vitamin P was added. The scratch areas of each group were recorded at different time points (0 h and 24 h), and the scratch closure rate was calculated via ImageJ software.

#### 3.2.6. Transwell Experiment

HCT116 and SW480 cells were cultured in serum-free DMEM supplemented with 0, 10, 20, or 40 μmol/L vitamin P and inoculated into the upper chamber of a Transwell plate (4 × 10^4^ cells per well). The Transwell system's lower chamber was supplemented with 10% FBS. Cotton swabs were used to remove all of the cells from the upper chamber of the culture plate after it had been incubated for 24 hours. PBS was used to wash the cells that had moved to the lower compartment, followed by 4% paraformaldehyde fixation and crystal violet staining. In order to count the cells, five areas were chosen at random under the microscope.

#### 3.2.7. Tumor Transplantation Experiments in Nude Mice

The animal experiments have obtained the corresponding production license number [SCXK (Sichuan) 2023-0040) and usage license number (SYXK (Sichuan) 2023-0263]. All BALB/c nude mice were raised in a specific pathogen-free (SPF) grade barrier environment to ensure aseptic conditions. During the breeding period, environmental parameters were precisely controlled: The room temperature was maintained between 26℃ and 28℃, the relative humidity was 50% to 60%, and a light-dark cycle of 10 hours of light and 14 hours of darkness was implemented. Animals could freely access drinking water and sterile standard feed that had been sterilized under high pressure. Before the formal experiment began, all nude mice received a one-week adaptive rearing to adapt to the environment and stabilize their physiological state. After completing adaptive feeding, we constructed a xenograft tumor model in nude mice by subcutaneous injection of colon cancer cell suspension. Specifically, human colorectal cancer cells in the logarithmic growth phase with good vitality were selected, digested with pancreatic enzymes, and suspended in a sterile PBS or matrix gel mixture. Then, a certain number of cells (for example, 5 × 10^6^ cells per cell) were inoculated subcutaneously under the right armpit or back of nude mice. Subsequently, the condition of the mice was closely observed and the tumor volume was measured regularly. When the tumors grew to a palpable size and moderate volume (usually about 100 mm³), the tumor-bearing mice were randomly grouped and intervention treatment with vitamin P or control solvent was initiated. After the treatment cycle ended, nude mice were euthanized under 2% isoflurane inhalation anesthesia (the specific method was cervical dislocation). Subsequently, the subcutaneous transplanted tumor tissue was rapidly dissected and completely excised. The surface bloodstains were gently rinsed with pre-cooled phosphate buffered saline (PBS), and the tumor weight was weighed using a precision balance and recorded. A portion of the excised tumor tissue was immediately immersed in 4% paraformaldehyde solution for fixation, and was used for subsequent paraffin embedding, sectioning, and various tests. These subsequent analyses included basic histological observations using conventional hematoxylin-eosin (H&E) staining, detection of the expression levels of target proteins such as Ki67 and VEGF using immunohistochemical (IHC) techniques, and detection of apoptosis within tumor cells by the TUNEL method, thereby comprehensively evaluating the anti-tumor efficacy of vitamin P at the in vivo level.

#### 3.2.8. Ki67 and Vascular Endothelial Growth Factor Expression in Tumor Tissues by Immunohistochemistry

The abdominal transplanted tumor tissues of the mice were fixed in 4% paraformaldehyde solution for 24 hours. After removal, the samples were paraffin embedded, sectioned, dried, dewaxed, and repaired with sodium citrate buffer, and anti-Ki67 (1:100) and anti-VEGF (1:100) antibodies were added to the samples, which were subsequently incubated at 4°C overnight. The expression of Ki67 and VEGF was observed under a fluorescence microscope. Positive expression of Ki67 is indicated by the appearance of brownish-yellow granular substances in the nucleus, and positive expression of VEGF is indicated by the appearance of brownish-yellow granular substances in the cytoplasm.

#### 3.2.9. TUNEL Was Used to Measure the Level of Apoptosis in Tissues

The transplanted tumor tissues from the abdomens of the mice were paraffin-embedded, sectioned, and dewaxed. Then, protease K was added for repair, and the endogenous peroxidase blocking solution was added for blocking for 5 minutes. After washing, DAB chromogenic solution was added for color development, and the cell nuclei were restained with hematoxylin.

### 3.3. Statistical Analysis

GraphPad Prism 9.0 was used for the statistical analysis. x±s is used to express all of the data. One-way analysis of variance was employed for comparisons of differences between several groups; when comparing two groups, the t test was used. If P was less than 0.05, the difference was deemed statistically significant.

## 4. Results

### 4.1. Vitamin P Inhibits the Viability of Colorectal Cancer Cells

**Figure 1.**
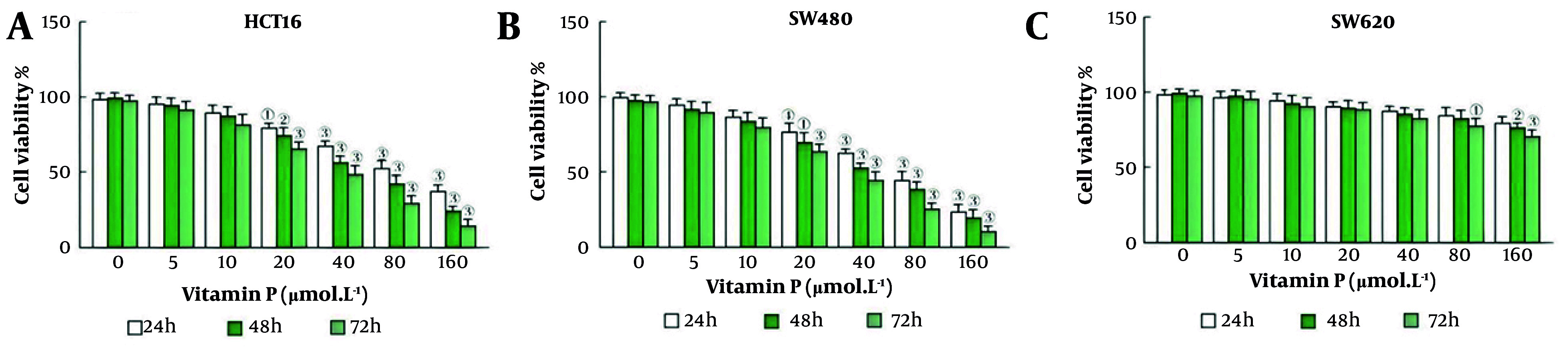
The impact of vitamin P on the viability of colorectal carcinoma cells; A, HCT116 cell viability; B, SW480 cell viability; C, SW620 cell viability (HCT116, N = 3, P = 0.002; SW480 cells, N = 3, P = 0.003; SW620 cells, N = 3, P < 0.001).

The results of the CCK-8 experiment revealed that vitamin P culture at a concentration of 20 μmol/L for 24 hours significantly inhibited the survival of HCT116 and SW480 cells (P = 0.002, P = 0.003; Figure 1A and B). However, the viability of SW620 cells significantly decreased only when they were treated with 80 μmol/L vitamin P for 72 hours (P = 0.002, Figure 1C). 

### 4.2. Vitamin P Inhibits the Colony Formation of Colorectal Cancer Cells

**Figure 2.**
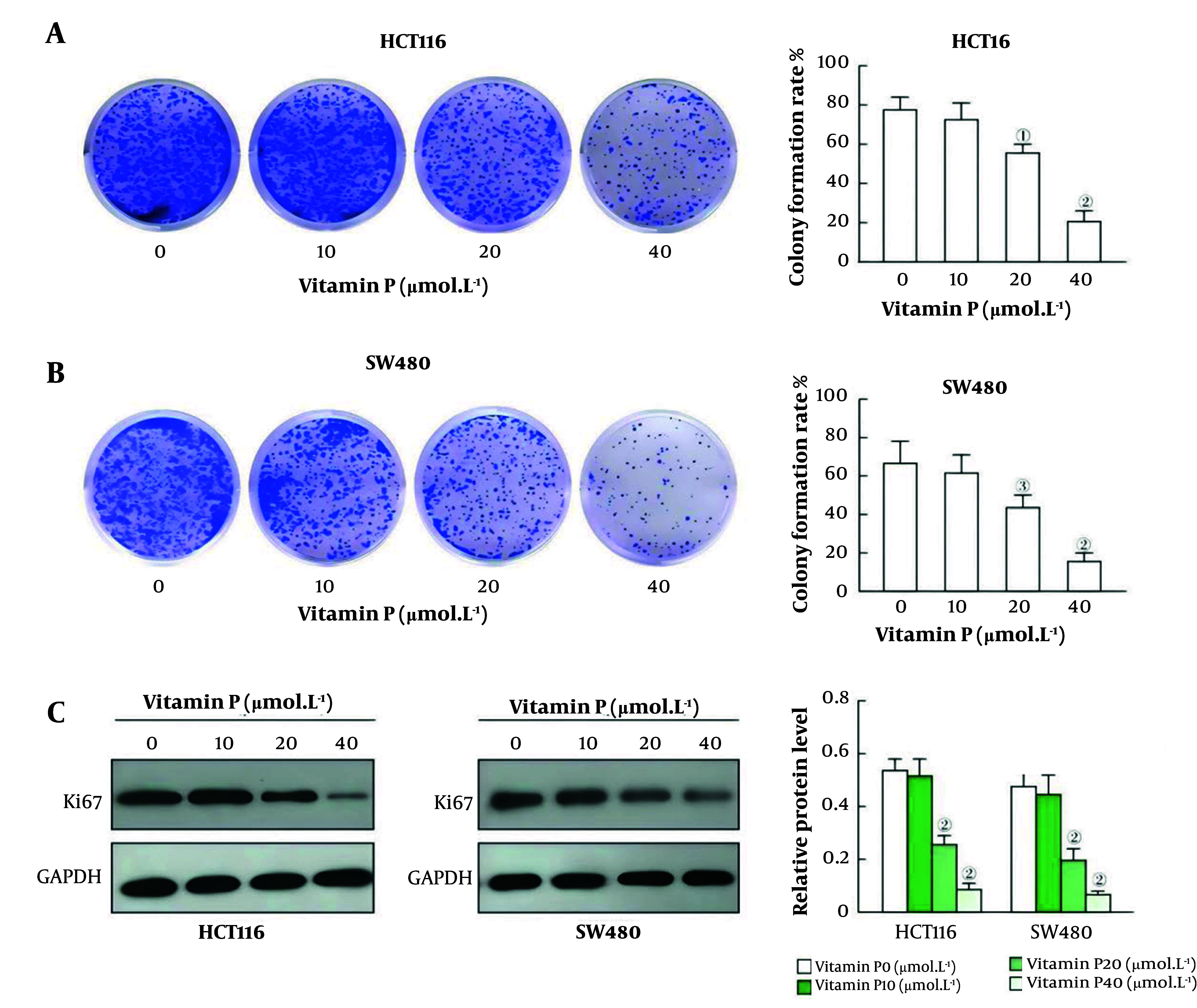
Effect of vitamin P on the colony formation of colorectal cancer cells; A, the colony formation rate statistics and representative pictures of HCT116 cell clones are presented in A; B, Average colony formation rate statistics and representative pictures of SW480 cell clones; C, Ki67 protein expression was detected by Western blotting, and the relative expression of Ki67 in HCT116 and SW480 cells was examined (HCT116, N = 3, P < 0.05; SW480 cells, N = 3, P < 0.05; SW620 cells, N = 3, P = 0.002).

The colony formation rates of the HCT116 and SW480 cells in the 20 μmol/L vitamin P group and the 40 μmol/L vitamin P group were significantly lower than those in the 0 μmol/L vitamin P group (all P < 0.05, Figure 2A and B). There was no statistically significant difference in the colony formation rate of the colorectal cancer cells between the 10 μmol/L vitamin P group and the 0 μmol/L vitamin P group (all P < 0.001, Figure 2C). 

### 4.3. Vitamin P Promotes the Apoptosis of Colorectal Cancer Cells

**Figure 3.**
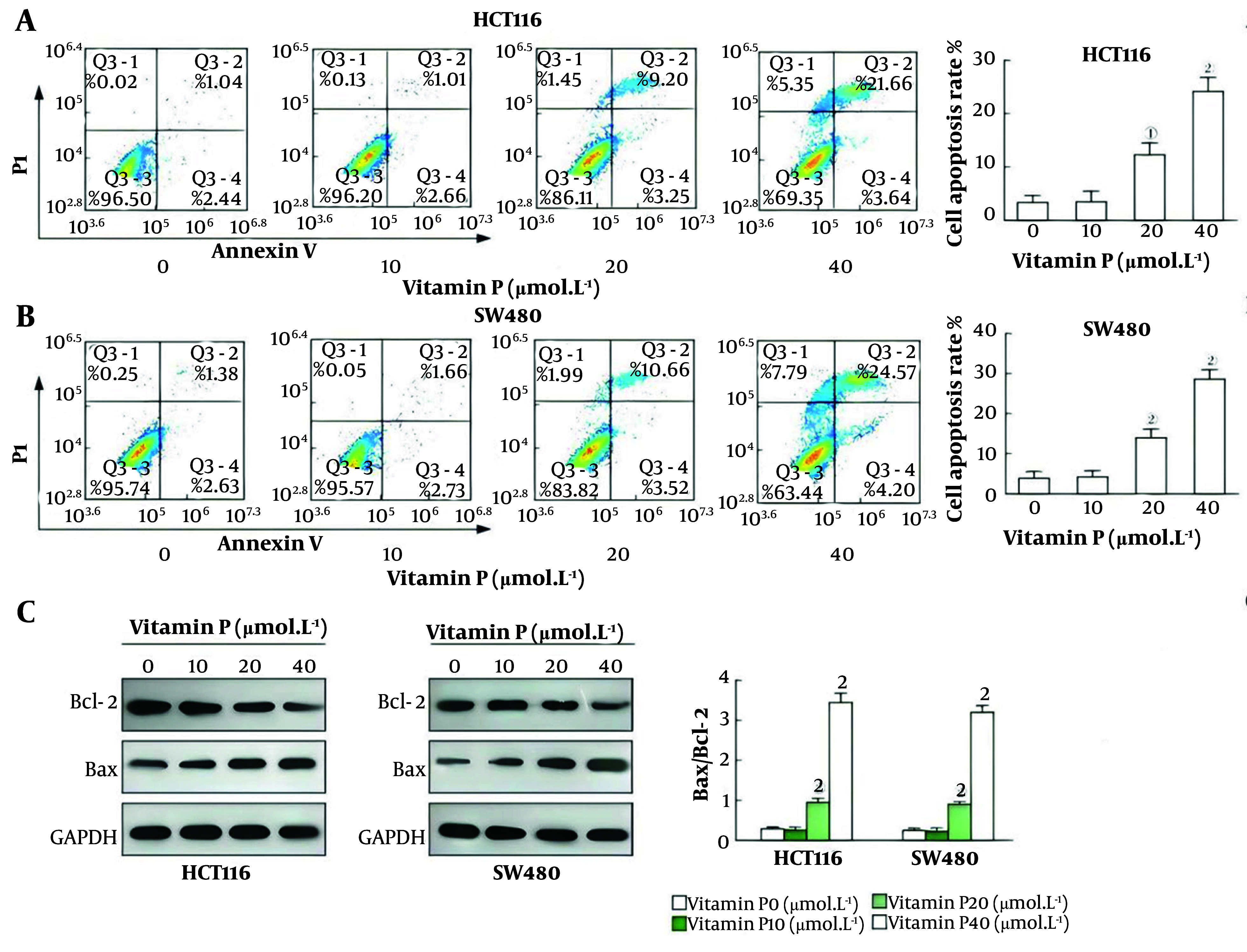
Effect of vitamin P on the apoptosis of colorectal cancer (CRC) cells; A, apoptosis was detected and the rate of apoptosis in HCT116 cells was investigated using flow cytometry; B, Using flow cytometry, apoptosis was identified, and the rate of apoptosis in HCT116 and SW480 cells was examined; C, Western blotting was used to identify the expression of the Bax and Bcl-2 proteins, and the Bax/Bcl-2 ratio in HCT116 and SW480 cells was computed (HCT116, N = 3, P < 0.05; SW480 cells, N = 3, P < 0.05; SW620 cells, N = 3, P < 0.05).

The apoptosis rates of the HCT116 and SW480 cells in the 20 μmol/L vitamin P group and the 40 μmol/L vitamin P group were significantly greater (all P < 0.05, Figure 3A and B). Compared with that in the 0 μmol/L vitamin P group, the ratio of Bax/Bcl-2 in the 20 μmol/L vitamin P group and the 40 μmol/L vitamin P group significantly increased (P < 0.05, Figure 3C). Nevertheless, there was no statistically significant variation in the Bax/Bcl-2 ratio in colorectal cancer cells between the 10 μmol/L vitamin P group and the 0 μmol/L vitamin P group.

### 4.4. Vitamin P Inhibits the Migration of Colorectal Cancer Cells

**Figure 4.**
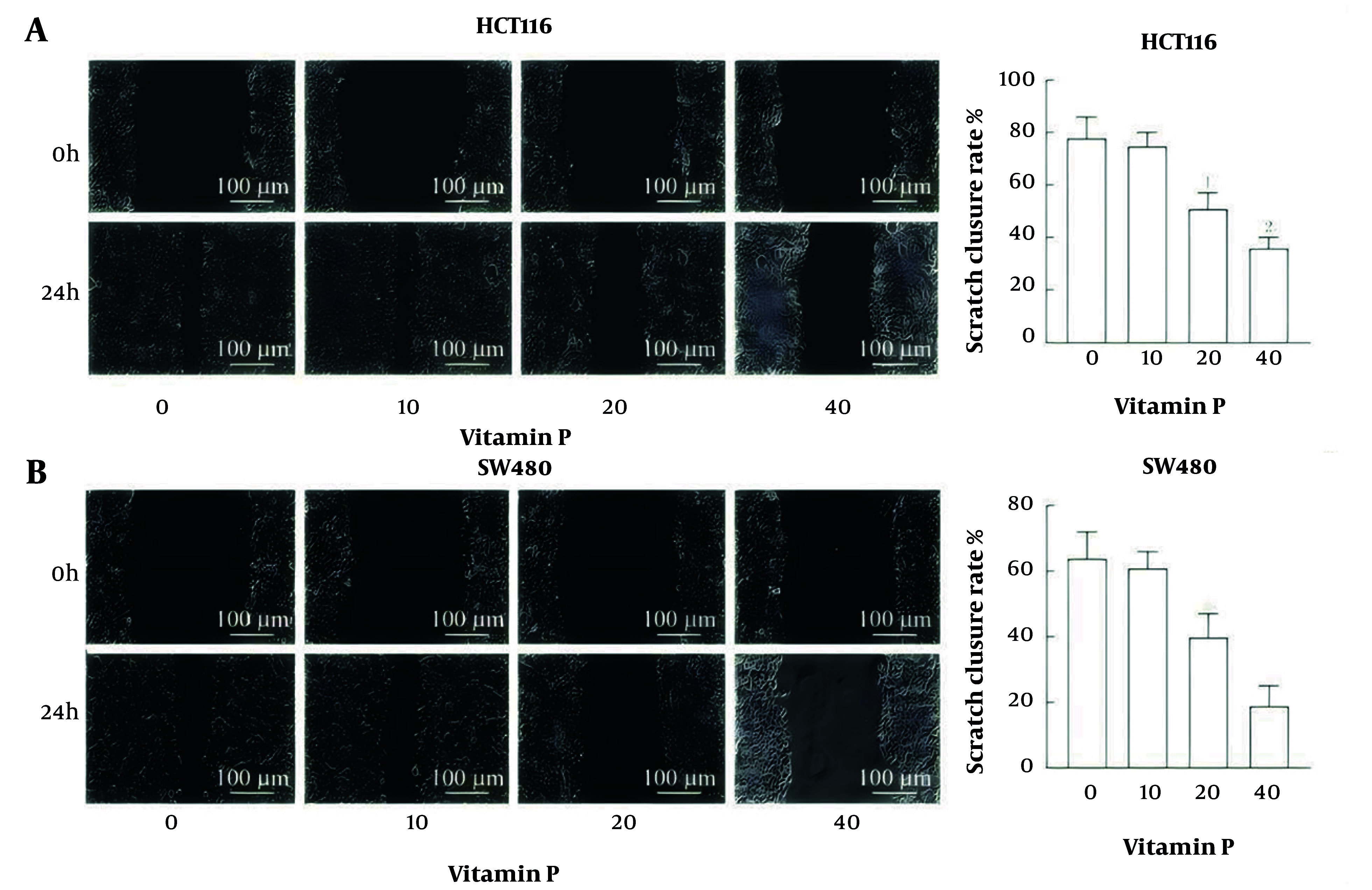
Effect of vitamin P on the migration of colorectal cancer cells; A, a scratch closure assay was used to evaluate migration ability, and the scratch closure rate of HCT116 cells was examined; B, the SW480 cell scratch closure rate was examined, and the migration capacity was evaluated via a scratch closure assay (HCT116, N = 3, P < 0.05; SW480 cells, N = 3, P < 0.05; SW620 cells, N = 3, P < 0.05).

The HCT116 and SW480 cells in the 20 μmol/L vitamin P group and the 40 μmol/L vitamin P group had much lower scratch closure rates than those in the 0 μmol/L vitamin P group (all P < 0.05, Figure 4). 

### 4.5. Vitamin P Inhibits the Invasion of Colorectal Cancer Cells

There were considerably fewer invading HCT116 and SW480 cells in the 20 μmol/L Vitamin P group and the 40 μmol/L Vitamin P group than in the 0 μmol/L Vitamin P group (all P < 0.001). However, there was no significant change in the number of invasive colorectal cancer cells in the 10 μmol/L Vitamin P group.

### 4.6. Vitamin P Inhibits the Growth of Colorectal Cancer

**Figure 5.**
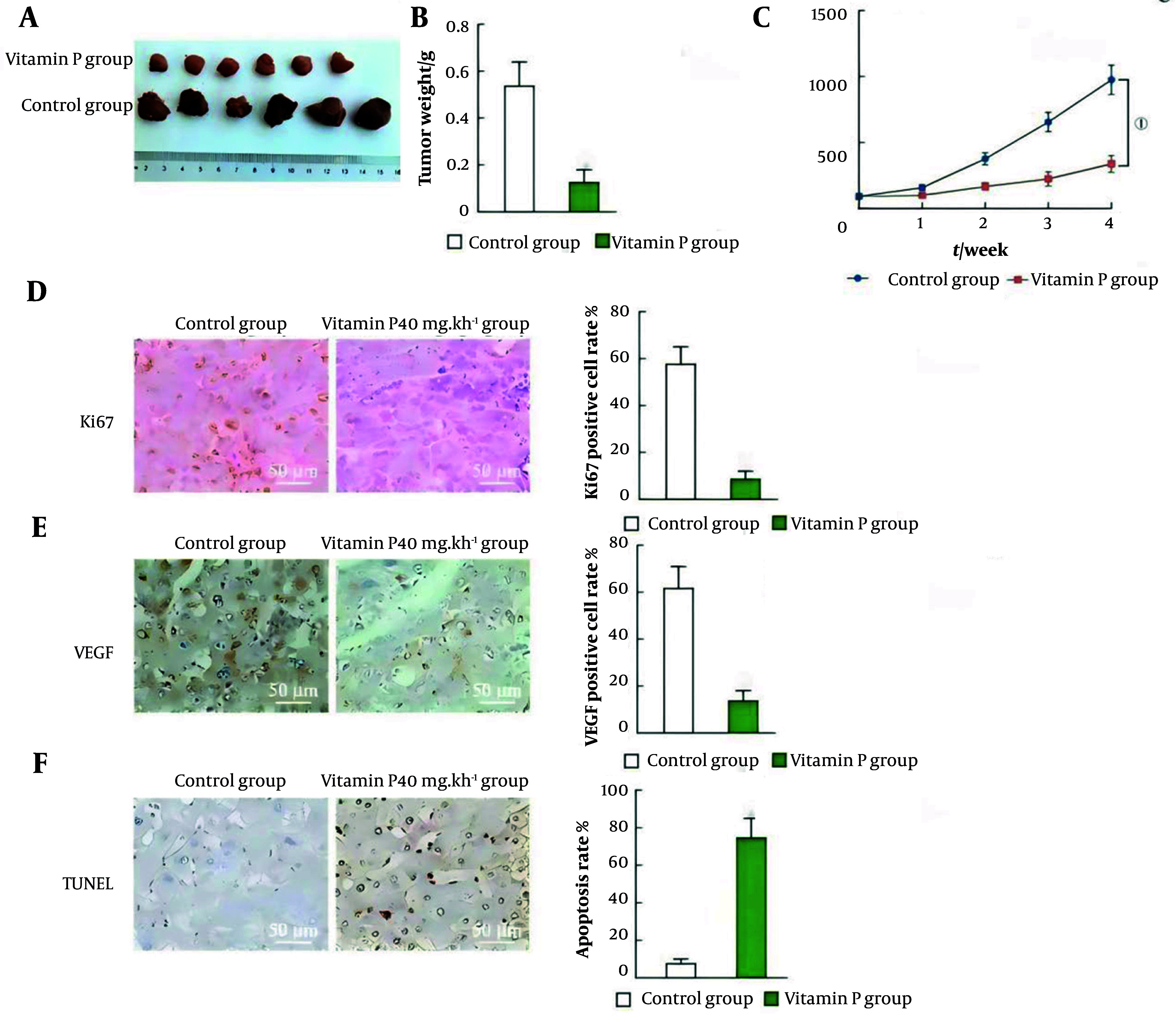
Effect of vitamin P on the growth of colorectal cancer xenograft tumors in vivo; A, tumor image; B, weight of the tumor; C, volume of the tumor; D, Ki67 expression was detected via immunohistochemistry, and the percentage of Ki67-positive cells was statistically analyzed; E, VEGF expression was determined via immunohistochemistry, and the percentage of VEGF-positive cells was statistically analyzed; F, apoptosis rate analysis and TUNEL detection of tumor cell apoptosis (mice: N = 6; HCT116, N = 3, P < 0.001; SW480 cells, N = 3, P < 0.001; SW620 cells, N = 3, P < 0.001).

The tumor mass and volume in the 40 mg/kg Vitamin P group were substantially smaller than those in the control group (P < 0.001; Figure 5A, B, and C), and the expression of Ki67 and VEGF was significantly lower (P < 0.001; Figure 5D and E). The TUNEL assay results revealed that the percentage of apoptotic tumor tissue cells in the 40 mg/kg vitamin P group was significantly greater (P < 0.001, Figure 5F). According to the findings of the histological examination of the mice's heart, liver, spleen, lung, and kidney, vitamin P did not appear to have any harmful effects.

## 5. Discussion

According to the findings of this study's cell-level research, vitamin P may stimulate apoptosis and prevent colorectal cancer cells from proliferating, migrating, and invading in vitro (13-15). It may also prevent tumor formation in established HCT116 ectopic and xenograft mice models (16). Inhibiting the excessive proliferation of tumor cells is the primary issue that needs to be addressed in the treatment of malignant tumors (17-19). We confirmed through the CCK-8 assay that when the concentration of vitamin P was above 20 μmol/L, vitamin P had a significant inhibitory effect on the proliferation of colorectal cancer cells (20). A colony formation assay confirmed that vitamin P could significantly inhibit the colony formation of colorectal cancer cells (21). The results of flow cytometry analysis confirmed that vitamin P could significantly promote the apoptosis of colorectal cancer cells. Ki67 is an indicator of the proliferation of cells. The higher its expression is, the more active the growth of tumor cells is (22-24). This is consistent with previously reported results of vitamin P-induced apoptosis of other tumor cells. Cell migration and invasion are key processes of cancer metastasis (25-27). The migration rates of both cell lines were inhibited, and vitamin P significantly reduced the number of invasive colorectal cancer cells (28-30). These results indicate that vitamin P can inhibit the migration and invasion abilities of colorectal cancer cells. Furthermore, the results of the tumorigenesis experiments in nude mice further verified the tumor growth inhibitory effect of vitamin P (31-33). Vitamin P (40 mg/kg) can reduce the size and weight of xenograft tumors and lower the expression of Ki67 and the vascular growth factor VEGF (34-38). In conclusion, this study confirmed vitamin P's ability to suppress colorectal cancer, providing new ideas for the effective development of vitamin P and the clinical treatment of colorectal cancer.

## Data Availability

The dataset presented in the study is available on request from the corresponding author during submission or after publication.
